# Dysregulated Expression of Transposable Elements in TDP-43^M337V^ Human Motor Neurons That Recapitulate Amyotrophic Lateral Sclerosis In Vitro

**DOI:** 10.3390/ijms232416222

**Published:** 2022-12-19

**Authors:** Braulio Valdebenito-Maturana, Matias Ignacio Rojas-Tapia, Mónica Carrasco, Juan Carlos Tapia

**Affiliations:** 1 Instituto de Investigación Interdisciplinaria, Vicerrectoría Académica, Universidad de Talca, Campus Talca, Talca 3460000, Chile; 2 Escuela de Medicina, Universidad de Talca, Campus Talca, Talca 3460000, Chile

**Keywords:** amyotrophic lateral sclerosis (ALS), TAR DNA binding protein 43 (TDP-43), transposable elements (TEs)

## Abstract

Amyotrophic lateral sclerosis (ALS) is a disease that progressively annihilates spinal cord motor neurons, causing severe motor decline and death. The disease is divided into familial and sporadic ALS. Mutations in the TAR DNA binding protein 43 (TDP-43) have been involved in the pathological emergence and progression of ALS, although the molecular mechanisms eliciting the disease are unknown. Transposable elements (TEs) and DNA sequences capable of transposing within the genome become dysregulated and transcribed in the presence of TDP-43 mutations. We performed RNA-Seq in human motor neurons (iMNs) derived from induced pluripotent stem cells (iPSCs) from TDP-43 wild-type—iMNs-TDP-43^WT^—and mutant—iMNs-TDP-43^M337V^—genotypes at 7 and 14 DIV, and, with state-of-the-art bioinformatic tools, analyzed whether TDP-43^M337V^ alters both gene expression and TE activity. Our results show that TDP-43^M337V^ induced global changes in the gene expression and TEs levels at all in vitro stages studied. Interestingly, many genetic pathways overlapped with that of the TEs activity, suggesting that TEs control the expression of several genes. TEs correlated with genes that played key roles in the extracellular matrix and RNA processing: all the regulatory pathways affected in ALS. Thus, the loss of TE regulation is present in TDP-43 mutations and is a critical determinant of the disease in human motor neurons. Overall, our results support the evidence that indicates TEs are critical regulatory sequences contributing to ALS neurodegeneration.

## 1. Introduction

Amyotrophic lateral sclerosis (ALS) is a lethal neurodegenerative disease whose causes and molecular faults are being elucidated. Spinal cord motor neurons (MNs) are undermined during the disease progression resulting in severe motor behavior decline, with muscle tone decay and weakness being the main cause of the respiratory failure observed in ALS patients [1]. Depending on heritability, the disease is classified into familial ALS (fALS)—which is passed on from one generation to the next—and sporadic ALS (sALS)—which is not inherited from parents but arises via mutations [2]. It has been estimated that almost all cases correspond to sALS, with only 5–10% corresponding to fALS [3,4,5,6,7]. Although more than 50 genes have been associated with fALS, about half of the cases have been attributed to mutations in four genes that encode (relative frequencies in parenthesis): *C9orf72* (chromosome 9 open reading frame 72, ~32%): *SOD1* (superoxide dismutase 1, ~11%), *TDP-43* (TAR DNA binding protein 43, ~4%), and *FUS/TLS* (Fused in Sarcoma/Translocated in LipoSarcoma, ~3%) [7,8]. The pathologic aggregation of TDP-43, a conserved nuclear RNA/DNA binding protein involved in the regulation of RNA processing (encoded by the *TARDBP* gene, chromosome 1), is a hallmark of both fALS and sALS, representing about 97% of all ALS cases [5]. Because of this, it remains at the core of the pathological proteins triggering the disease [6]. TDP-43, in addition to the regulation and estimation of ~6000 mRNAs (~30% of the human transcriptome) controls several aspects of RNA metabolism, i.e., transcription, translation, maturation, transport, stability, and localization [6]. Given the ability to act as a master regulator, it is not surprising that TDP-43 has been involved in ALS. In fact, one of the characteristic features of ALS is the accumulation of ubiquitin-immunoreactive cytoplasmic inclusions in damaged motor neurons; inclusions that show the aggregates of TDP-43 with abnormal C-terminal fragments that are highly ubiquitinated and phosphorylated [7,8]. More than 50 mutations have been found in ALS patients, with TDP-43^M337V^ being one of the most common [5,6,9]. Unfortunately, the link between TDP-43 alterations and ALS neurodegeneration continues to be undetermined, and a significant effort is being put into elucidating the molecular pathways altered by TDP-43 mutations.

Transposable elements (TEs), also known as mobile genes, are DNA sequences that make up about half of all the sequences in the human genome and can move from one location to another within it. Based on the transposition mechanisms, TE sequences have been classified into retrotransposons (class I)—mobilize through an RNA intermediary generated via reverse transcription, and DNA transposons (class II)—and are directly excised from the genome (i.e., do not use RNA as an intermediary) [10]. Retrotransposons are further subclassified into long terminal repeats (LTRs) and non-LTRs [11]. Non-LTR TEs comprise long interspersed nuclear elements (LINEs) and short interspersed nuclear elements (SINEs) [10,11]. Although most TEs in the human genome are unable to transpose, extensive evidence indicates that they become transcriptionally active and thus can regulate gene expression [12]. Interestingly, upon TDP-43 alterations, LTR and non-LTR TEs transcripts are highly dysregulated [13], suggesting that TEs activity contributes to TDP-43- related ALS. 

The activation of TEs in other neurodegenerative diseases has been reported previously. In Tau-associated pathologies, such as supranuclear palsy and Alzheimer’s disease (AD), TEs can become activated [14,15]. These works point to the role of TEs in contributing to neurodegeneration and DNA damage. A similar finding has been reported for TE activity in Parkinson’s disease (PD): the expression of the L1 LINE TE occurs as a consequence of oxidative stress and results in DNA damage, which in turn causes neuron death. Interestingly, in this work, the authors were able to pinpoint a functional role in this, in which by blocking L1 expression, neuron death was decreased [16]. Previously, it has been reported that AD and PD have commonalities with ALS in terms of the molecular pathways afflicted [17]. A similar finding has been seen for ataxia telangiectasia, in which there seems to be an interplay between DNA damage and L1 TE activity. However, the authors speculated that the functional contribution of TEs still needs to be elucidated in order to discern whether they are a cause or a consequence of the disease [18]. For Rett syndrome (RTT), a similar outcome has been reported in that, although L1 activity can be observed because of the loss in DNA methylation patterns, it is yet to be determined if this corresponds to a causal contributor of the disease [19]. More recently, in a model of Huntington’s disease (HD), it was shown that the retrotransposition of TEs contributed to neuronal toxicity and DNA damage, and through the repression of the retrotransposition event, these effects could be reversed [20]. Collectively, these reports highlight that TE activity is highly intertwined with neurodegenerative diseases. 

As the TDP-43 malfunction is common to both sALS and fALS, and considering that TDP-43 modulates TE expression [13,21], changes in the TE activity upon ALS can also occur. Indeed, one of the first reports pointing to TE activity in ALS revealed the transcription of human endogenous retroviruses (HERVs): a type of LTR TE, in ALS patients [22]. There is evidence showing that in the *Drosophila melanogaster* model of TDP-43, TEs become activated [23]. On the other hand, we have shown that in the SOD1^G93A^ mouse model of the disease, TEs can be activated and might influence gene regulation across the disease progression [24]. Two works have revealed that in brains obtained from postmortem ALS patients, TEs become activated [13,25]. Interestingly, in the work of Tam et al. [13], they were able to validate that TDP-43 did indeed repress TEs because, through a knockdown experiment, an increase in TE activity was seen. This is in line with a previous work highlighting the relationship between TDP-43 and TEs in a murine TDP-43 ALS model [21]. Although there is a big body in the literature pointing to the activation of TEs in ALS and other neurodegenerative diseases, a caveat is that they did not use RNA-Seq data and/or those that applied the technique and did not measure the locus-specific activity of TEs (except for our previous report in the SOD1^G93A^ mouse model) using the novel bioinformatic tools that allowed this to be conducted.

Induced pluripotent stem cells (iPSCs) are derived from human somatic tissues and offer advantages in terms of the availability of modeling diseases, drug discovery, and providing promising therapeutical approaches for applications in personalized medicine [26,27]. Thus, we established an in vitro model of mutant TDP-43 ALS based on induced pluripotent stem cells (iPSCs) with the goal of understanding the mechanisms underlying ALS [28]. The model consisted of induced motor neurons (iMNs) derived from iPSCs obtained from healthy, i.e., wild-type (WT), and ALS patients carrying one of the most common mutations in TDP-43 (MT TDP-43^M337V^). The iMNs-TDP-43^M337V^ model recapitulated several hallmarks of the ALS disease in vitro. For example, at ~7 days in vitro (DIV), iMNs-TDP-43^M337V^ showed cytoplasmic inclusions with delocalized TDP-43, fragmented neurites, altered cytoskeleton, mitochondrial swelling, etc. These pathological signs were more frequent at 14 DIV, affecting the physiological properties of iMNs-TDP-43^M337V^ but not the control cells [28]. To understand the molecular changes that took place at these stages, we profiled iMNs-TDP-43^WT^ and iMNs-TDP-43^M337V^ at 7 and 14 DIV with RNA-Seq and studied the changes in gene expression and TEs activity. We analyzed diverse gene pathways with the disease progression in vitro by either directly studying changes in the gene expression or by indirectly associating TEs activity with altered gene programs. Overall, we found extensive changes in both genes and TEs. Interestingly, several gene programs had a strong association with TEs levels, suggesting that the putative transcriptional control becomes dysregulated in the presence of TDP-43^M337V^. The TEs-gene pairs can be associated with several biological processes involved in ALS pathogenesis. In sum, we conclude that TEs are central to TDP-43^M337V^-induced ALS disease.

## 2. Results

### 2.1. Differential Expression of Genes and Transposable Elements in the Mutant iMNs

**Figure 1:**
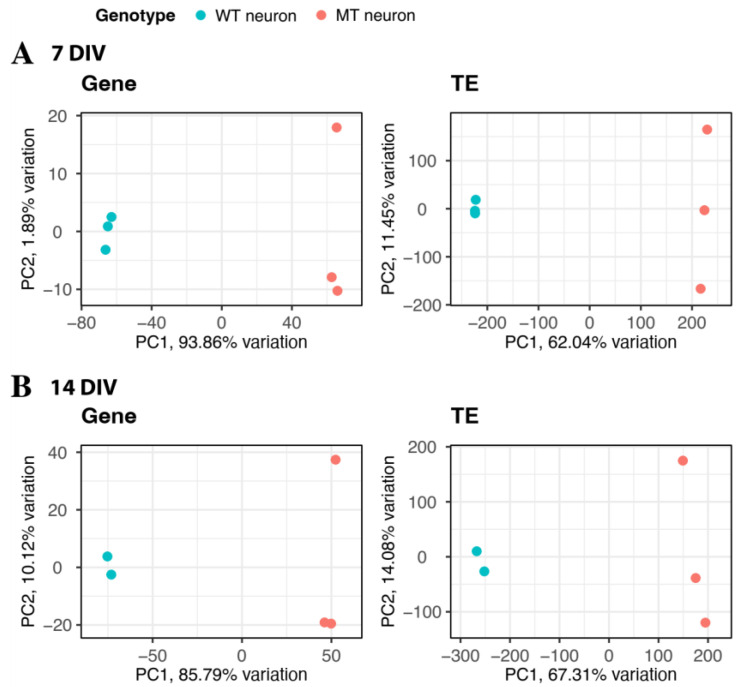
PCA analysis of RNA-Seq libraries for genes and TEs (**A**) PCAs corresponding to genes (**left**) and TEs (**right**) at 7 DIV (**top** panels). (**B**) 14 DIV (**bottom** panels) results are shown. iMNs TDP-43^WT^ and iMNs TDP-43 ^M337V^ samples are shown in blue and red, respectively. Despite the inherent expression variance, samples can be clustered as indicated by PCAs.

**Figure 2:**
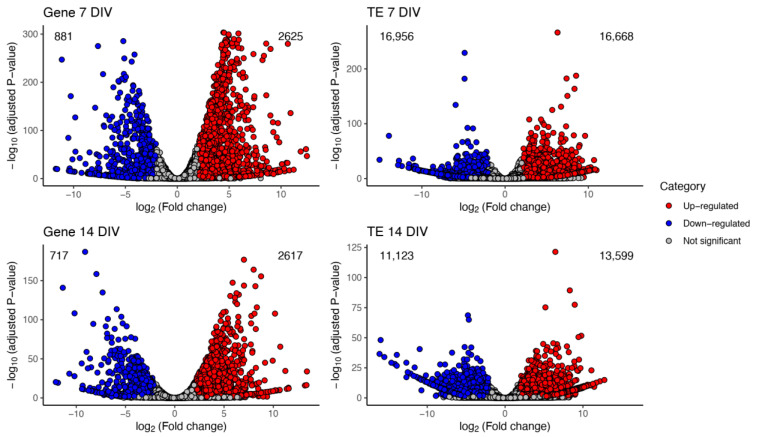
Differential expression of genes and TEs in iMNs TDP-43^WT^ and TDP-43^M337V^. Volcano plots showing all genes (**left**) and TEs (**right**) differentially expressed (DE) at 7 DIV (**top** panel) and 14 DIV (**lower** panel). Up-regulated and down-regulated genes and TEs are shown in red and blue circles; gray circles represent genes and TEs that did not meet the overall expression threshold (|log_2_(fold change)|≥ 2; adjusted *p*-value ≤ 0.05; see Section 4).

We performed RNA-Seq on iMNs WT and MT- TDP-43^M337V^ samples obtaining 100bp paired-end reading with an average number of reads per library of 55,344,184 (see Section 4). Three replicates per condition were obtained, except for the 14 DIV WT sample, which was discarded due to poor quality. RNA-Seq libraries were aligned to the human genome, and both the gene and TE expression were analyzed (Methods). As a first step to assess how closely related the samples were to each other, we performed a Principal component analysis (PCA; Methods) of the expression profiles and evaluated whether the genes (Figure 1, left) and TEs (Figure 1, right) explained the sample differences at 7 DIV (Figure 1, top panels) and 14 DIV (Figure 2, bottom panels).

According to PCA analysis, similar sample clusters, i.e., iMNs TDP-43^WT^ vs. iMNs TDP-43^M337V^, were present when the gene or TE expression was quantified. Even though there were fewer variances among the TEs, we observed that iMNs TDP-43^WT^ were consistently differentiated from the iMNs TDP-43^M337V^ samples and were always clustered within the same groups (Figure 1).

We then performed differential expression (DE) analysis at 7 and 14 DIV between the iMNs-TDP-43^WT^ and iMNs-TDP-43^M337V^ genotypes to obtain a global view of the number of genes and TEs whose expression profiles changed between the genotypes. Statistically significant changes in expression were found for 3506 and 3334 genes at 7 and 14 DIV, respectively (Table 1, Figure 2, Supplementary File S1). Nearly ~75% of the genes were quantified corresponding to the up-regulated genes and meaning genes whose expression values were higher in iMNs TDP-43^M337V^ with respect to iMNs TDP-43^WT^.

We also quantified TEs levels in iMNs TDP-43^M337V^ and compared them to iMNs TDP-43^WT^. In this case, we found larger numbers of differentially expressed TEs at 7 DIV than at 14 DIV; nearly 33,624 TEs were found at 7DIV, while 24,722 TEs changed expression levels at 14 DIV, both of which were distributed in equivalent proportions between the up-and down-regulated groups.

To add further support to our results, we compared the DE genes to those previously reported by Tam et al., (2019) [13]. Briefly, in their work, they profiled the transcriptomes of postmortem brains obtained from ALS patients, and based on gene expression, they classified the transcriptomes into three groups: ALS-TE (increased transposable element expression), ALS-Ox (increased expression of oxidative stress genes), and ALS-Glia (increased expression of glial genes). Overall, we found a match between ~120 of our DE genes with those reported by Tam et al. [13] (Supplementary File S2). We performed a similar comparison for TEs, but only at the subfamily level, as the authors did not study TEs with a locus-specific resolution. In this regard, out of the six subfamilies reported, we found a match with our results in three of them.

Collectively, our results indicate that both genes and TEs undergo extensive-expression changes during disease progression in iMNs TDP-43^M337V^ with respect to iMNs TDP-43^WT^. To better understand the large-scale impact of such changes, we sought to identify through gene enrichment analysis the genetic pathways altered in TDP-43^M337V^ ALS.

### 2.2. Genetic Programs Associated with Differentially Expressed Genes

**Figure 3:**
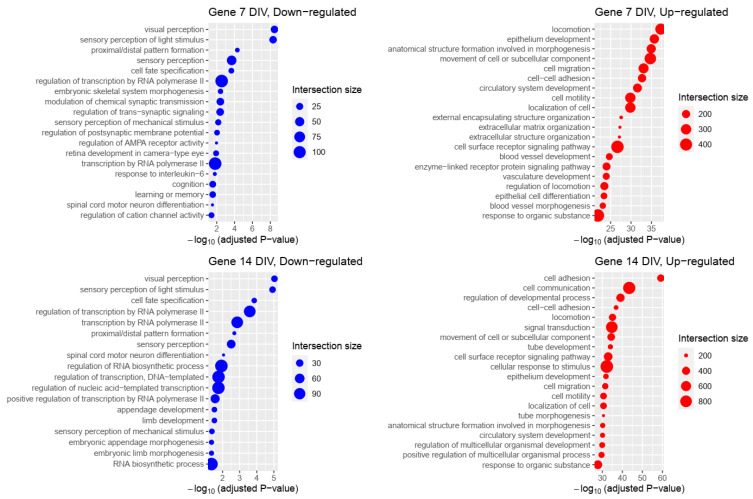
Biological process enrichment analysis of differentially expressed genes in iMNs TDP-43^M337V^. Top 20 enriched biological processes associated with differentially expressed (DE) genes, down-regulated (blue) and up-regulated (red) at each time point are shown. Each list goes in descending order according to their statistical significance. The dot size corresponds to the number of genes associated with the respective biological process (“Intersection size”).

Gene ontology (GO) enrichment analysis was used as a method to identify the gene annotations that participated in the relevant biological processes. Thus, GO annotations were performed in down-regulated and up-regulated genes from iMNs TDP-43^M337V^ vs. iMNs TDP-43^WT^ genotypes at 7 and 14 DIV to identify the key biological processes (Figure 3, Supplementary File S3). 

The results showed that the down-regulated genes were associated with various biological processes, including spinal cord motor neuron differentiation and visual and sensory perception, which correlated with clinical symptoms of the disease. We also observed the enrichment of genetic pathways involved in cognition and memory. Not surprisingly, several gene processes associated with RNA metabolism were negatively enriched, in line with the consequences of TDP-43 dysfunction and downstream changes in gene regulation. Of relevance to ALS, spinal cord motor neuron differentiation appeared to be negatively regulated, pinpointing defects in the primary cell type afflicted by the disease.

On the other side, for differentially up-regulated genes, a positive association was found for the biological processes, which included cell adhesion, cell communication, locomotion, and cell surface receptor signaling pathways. This is in line with the evidence indicating that TDP-43 pathologic aggregates spread from cell to cell [29]. For both the up-and down-regulated genes, strong associations were also found with biological processes involved in RNA regulation, synthesis, and metabolism. Additionally, we compared the genes belonging to each of the enriched processes to the known ALS genes reported in [30]. We were able to find that *ANXA11* (Annexin A11) appears in five statistically significant GO clusters at 7 DIV: *“response to stimulus”, “response to chemical”, “localization”, “transport”, and “establishment of localization”*. This gene was later associated with ALS in another independent work [31]. We also noted some agreement with previous studies [32,33] in cell adhesion and blood vessel-related terms. In sum, differential expression gene analysis confirms several of the biological processes that have been highlighted in ALS.

### 2.3. Impact of Transposable Elements in Genetic Programs

To gain further insights into the consequences of such changes, we correlated TE expression to gene expression using a two-fold statistical approach (see Section 4). First, we associated each TE with the gene located closest to them in the genome and then applied a linear model in which gene expression was modeled as a function of TE expression. The approach allowed us to determine genes that were likely regulated by TE levels. Then, the correlation between the expression of each gene-TE pair was calculated to assess whether the TEs positively or negatively impacted gene expression.

**Figure 4:**
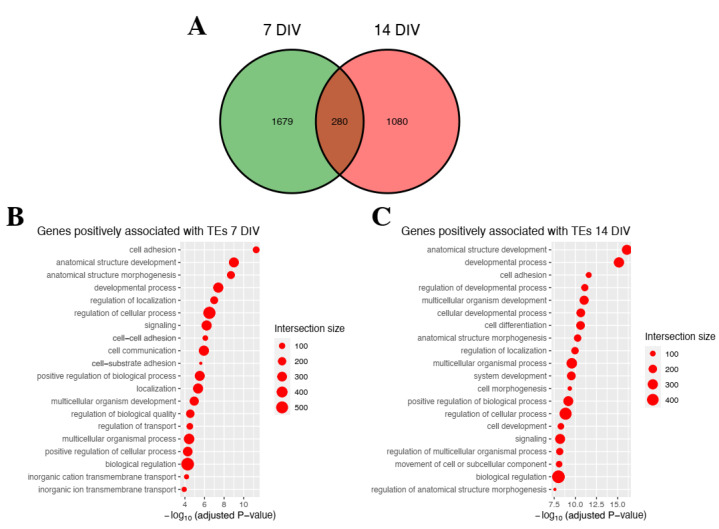
Pathway enrichment analysis for genes associated with differentially expressed TEs iMNs TDP-43^M337V^. (**A**) A Venn diagram is shown to indicate the number of gene-TE pairs associated with each time point: 7 (green), and 14 DIV (red). (**B**) Top 20 enriched biological processes of genes positively associated with TEs at 7 DIV. (**C**) Top 20 enriched biological processes of genes positively associated with TEs at 14 DIV. Each list goes in descending order according to their statistical significance. The dot size corresponds to the number of genes associated with the respective biological process (“Intersection size”; see Section 4).

About 1959 (1873 positive, 86 negative) gene-TE pairs were obtained at 7 DIV and 1360 (1324 positive, 36 negative) at 14 DIV, with 280 gene-TEs pairs appearing common to both time points (Figure 4). 

Similar to the previous result, we performed an enrichment analysis of the genes associated with the TEs following the aforementioned protocol in order to determine the biological processes involved in which TEs could be impacting. We did not obtain significant (adjusted *p*-value ≤ 0.05) GO enrichment of the genes negatively correlated with TEs at either stage. This is probably due to the small number of those genes (86 at 7 DIV, 36 at 14 DIV) impairing the enrichment analysis. For the positively correlated gene: TE pairs and associations to biological processes that included cell communication, cell adhesion, localization and signaling, and morphogenesis were highly enriched. Overall, the results suggested that TEs contributed to the changes in gene expression involved in iMNs ALS, which negatively impacted the viability of iMNs contributing to the disease progression.

## 3. Discussion

In the present study, we sequenced and analyzed RNA-Seq datasets from wild-type (TDP-43^WT^) and mutant (TDP-43^M337V^) iMNs at 7 and 14 DIV to identify differentially expressed genes and, importantly, TEs. Significant changes in both the gene expression and TEs activity were shown, indicating that in TDP-43 mutants, both the genes and TEs were highly dysregulated. In turn, based on gene enrichment analyses, several genetic programs were altered in the iMNs ALS model, which was consistent with previous reports. Interestingly, gene-TE pairs were associated with several cell biological processes that might contribute directly to the disease pathology. For example, biological processes that impacted and could be linked to ALS were cellular morphogenesis, visual and sensory perception, locomotion, cognition, and memory, RNA synthesis, processing, and metabolism; this is in line with the consequences of TDP-43 dysfunction and the downstream changes in gene regulation. Of relevance to ALS, spinal cord motor neuron differentiation seems to be negatively regulated, pinpointing the defects that occur in the primary cell types afflicted by ALS. Additionally, important previous works have indicated that cell adhesion and extracellular matrix components are essential for maintaining normal cellular functions and play critical roles in axonal growth, myelination, synaptogenesis, cytoskeleton, and intercellular signaling. It is unclear whether the enrichment pathways indicated by gene-TE pairs are primary to the ALS pathology or result as a consequence of downstream alterations. In general, our differential gene expression profiles are consistent with the findings reported by Lin et al. [32] and Kotni et al. [33], in which both analyzed gene expression in different ALS models. 

We and others have previously reported the activation of TEs in ALS [13,24,34]. Here, using iMNs, a different model than the one used in those works, we were also able to find that TEs undergo expression changes in the disease. These elements, in turn, highlight the importance of studying and suggests that their expression might be more intertwined with the disease. Although there is still more to elucidate regarding the role of TEs in ALS, our work presents additional evidence to suggest the role of TEs during ALS progression by modulating gene expression. Future works are needed to further understand whether TE-mediated regulation is a cause or a consequence of the disease.

In sum, our gene and TEs expression analysis in iMNs mutants (TDP-43^M337V^ with respect to TDP-43^WT^) confirm the presence of thousands of TEs; these differentially expressed TEs could potentially play a role in regulating the expression of genes that participate in several biological processes relevant for ALS pathogenesis and progression. 

## 4. Materials and Methods

RNA-Seq analyses were carried out from patient-derived iPS cells (wtTDP43, mtTDP43-M337V). We cultured the differentiated motor neurons on glass coverslips that were inverted over a monolayer of mouse cortical glia separated by a sterile paraffin tripod. RNA-sequencing was performed on an Illumina HiSEQ, with a paired-end layout, generating 2 × 100 bp reads to a depth of 50 million reads on average.

The RNA-Seq reads were aligned using STAR v2.7.6 [35] to the human genome hg38 version obtained from UCSC. The reads per gene were quantified using feature Counts, and the hg38 GTF annotation file was also obtained from UCSC. The locus-specific quantification of transposable elements expression was performed with a Telescope [36]. Transposable elements fully contained within exons were not considered for further analysis.

Differential expression analysis was conducted with DESeq2 [37], using the wild-type samples as the controls. Genes and TEs with |log_2_(Fold change)|≥ 2 and adjusted *p*-value ≤ 0.05 were considered to be differentially expressed.

BEDtools [38] was used to identify the closest gene within 5000 bp of each DE TE and were obtained at 7 DIV and at 14 DIV. With this information, we built a table of gene-TE pairs, which were then used as input for TEffectR [39] to model the gene expression as a function of TE expression and identify statistically significant associations in which TE expression might explain the changes in gene expression. Gene-TE pairs with a model *p*-value ≤ 0.05 were selected, and the correlation between them was calculated with the “cor” [37] function of the R statistical computing language [40]. We then kept only those results that had an absolute correlation value ≥ 0.8. From these results, we generated a list of genes that were significantly associated with DE TEs, which was also used for gene enrichment analysis (described next).

Gene enrichment analysis was performed using the g:Profiler web server [41]. We used the DE genes at 7 DIV and at 14 DIV as the input, and also those genes associated with TEs, following the protocol described in the previous paragraph. Biological processes with a significant enrichment with adjusted *p*-value ≤ 0.05 were considered.

All plots were conducted with ggplot2 [42], and the Venn diagrams with ggvenn (https://cran.r-project.org/web/packages/ggvenn/, accessed on 1 November 2022).

## 5. Conclusions

Our results indicate that TEs are central to TDP-43^M337V^ ALS disease progression. Several genetic programs are modulated by the dysregulation of TEs and by the mutated form of TDP-43. Thus, TEs become a key pathological factor for the pathogenesis and progression of the disease. 

## Figures and Tables

**Table 1:** Number of differentially expressed genes and TEs across each time point.

Timepoint	Up-Regulated Genes	Down-Regulated Genes	Up-Regulated TEs	Down-Regulated TEs
7 DIV	2625	881	16,668	16,956
14 DIV	2617	717	13,599	11,123

## Supplementary Materials

The following supporting information can be downloaded at: https://www.mdpi.com/article/10.3390/ijms232416222/s1, Supplementary File S1: Differential expression analysis; Supplementary File S2: Comparison with Tam et al., (2019) [13] ALS postmortem dataset; Supplementary File S3: Gene enrichment analysis.
